# Management of Craniopharyngioma – Perspectives beyond Surgery and Endocrinology

**DOI:** 10.17925/EE.2015.11.02.96

**Published:** 2015-08-19

**Authors:** Rachel K Crowley, Christopher J Thompson

**Affiliations:** 1. Consultant Endocrinologist, St Vincent’s University Hospital, Elm Park, Dublin, Ireland; 2. University College Dublin, Dublin, Ireland; 3. Consultant Endocrinologist, Beaumont Hospital, Dublin, Ireland

**Keywords:** Craniopharyngioma

## Abstract

The excess mortality in craniopharyngiomas is attributable to their size, site and the traditional surgical approach; aggressive resection predisposes to hypothalamic complications such as obesity, somnolence, thirst disorders and neurocognitive dysfunction. Recently, treatment has been modified to partial resection and radiotherapy. The role of the endocrinologist has expanded from identification and replacement of hormone deficits to include management of hypothalamic disease. Future treatment of craniopharyngioma with neo-adjuvant chemotherapy to minimise surgical resection may improve the outcomes for these patients.

Craniopharyngiomas are large tumours that extend beyond the sella and suprasellar region into the hypothalamus.^1^ Every case series has indicated a higher mortality in craniopharyngioma than has been reported in pituitary adenoma series or in other cases of hypopituitarism. There is now a better understanding of the factors that contribute to both the morbidity and mortality associated with craniopharyngioma, which has led to a change in clinical practice and an expansion of the role of the endocrinologist.

## Traditional Approach to Management of the Patient with Craniopharyngioma

Although the histology is described as benign, craniopharyngiomas form micropapillary extensions into surrounding hypothalamic tissue, which promote a gliotic reaction.^2^ Surgical resection of these lesions is difficult because of their adherence to surrounding hypothalamic structures. The traditional surgical approach was to attempt gross total resection (GTR), often via a frontal or pterional craniotomy. Aggressive surgery was associated with high rates of panhypopituitarism and diabetes insipidus, morbid obesity, disorders of appetite and thirst, somnolence, poikilothermia and a range of neurocognitive deficits.^3–8^ Despite attempts at surgical clearance, rates of tumour recurrence were as high as 40 %.^3^

Traditional methods of management of craniopharyngioma are also associated with high standardised mortality, despite adequate replacement of pituitary hormone deficiencies.^3,9,10^ Routine management has changed to address some of the morbidities associated with craniopharyngioma.

## Current Approach

In the last 10 years there has been a change in surgical approach to a modified transsphenoidal resection in parallel with radiotherapy and radiosurgery. It is not possible to conduct large-scale trials for this rare tumour, but published data indicate that less-aggressive surgery is not complicated by an increase in tumour recurrence rates.^11,12^ Hypothalamus-sparing surgery in children with craniopharyngioma results in lower rates of obesity than in children who have undergone a traditional surgical approach.^11^

Analysis of large pituitary disease registers has revealed that patients with craniopharyngioma differ from other hypopituitary patient groups in the rates of hormone deficiency and response to replacement therapy. Data show that they have higher rates of pre-receptor activation of cortisone to cortisol by the enzyme 11βHSD1.^13^ There may be less benefit from growth hormone (GH) replacement therapy in reduction of visceral adiposity and improved lipid profile.^14^ Although GH replacement does not appear to reduce the mortality associated with craniopharyngioma, GH therapy is safe for these patients and does not lead to tumour recurrence.^15^ Craniopharyngioma is also associated with higher rates of diabetes insipidus that may be complicated by adipsia or polydipsia.^16^ It is unclear whether modification of hormone replacement therapy will reduce the morbidity and mortality associated with craniopharyngioma; however, the recognition of the unique abnormalities seen in craniopharyngioma patients does prompt the need for further research into accurate hormone replacement in this cohort.

Better recognition of hypothalamic complications of craniopharyngioma has led to more active management of a number of conditions. Obesity is multifactorial and treatment should encompass a personalised approach. Bariatric surgery may be useful in some patients; a metaanalysis of a study and a number of case reports have shown that such an approach can be effective and safe in halting or reversing weight gain in craniopharyngioma patients with obesity.^17^ The concerns about bariatric surgery must be carefully weighed against the complications of obesity, on an individual basis. Obesity in craniopharyngioma has also been treated successfully with exenatide (12.8 % weight loss),^18^ and by the combination of diazoxide and metformin (stabilisation of weight gain).^19^ It is likely that early intervention to prevent obesity will be most effective; once hypothalamic obesity is established both somnolence and immobility render it difficult to treat. There is a spectrum of sleep disorders in craniopharyngioma including obstructive sleep apnoea (OSA), somnolence and narcolepsy and all exert a negative impact on quality of life. Diagnosis of the underlying sleep disorder is essential for appropriate treatment; OSA may respond to continuous positive air pressure (CPAP) therapy^4^ and somnolence may respond to melatonin or modafinil.^4,20^ Management of thirst disorders is complex; monitoring for dysnatraemia identifies those with hypernatraemic dehydration who require intravenous rehydration and anticoagulation to prevent thrombosis.^16^ Active management of hypothalamic disease has led to an improvement in quality of life for many craniopharyngioma patients; we routinely screen for sleep and thirst disorders, and adopt a preventative approach to obesity.

## Potential Future Therapy

The ideal therapeutic tool for the team managing craniopharyngioma patients in the future would be a chemotherapeutic agent that could shrink craniopharyngioma tissue away from the hypothalamus and allow a surgical resection without damage to hypothalamic nuclei. The identification of a BRAF mutation in the papillary subtype of craniopharyngioma has revealed an exciting potential target for a chemotherapeutic approach.^21^ BRAF inhibitors are already in clinical use for melanoma, and a case of response to a BRAF inhibitor, vemurafenib, in a craniopharyngioma patient has been reported.^22^ A formal clinical trial of this drug class in craniopharyngioma is in development.

It is unlikely that neoadjuvant chemotherapy for craniopharyngioma will entirely abolish hypothalamic complications of this disease, therefore individualisation of the approach to hypothalamic complications in craniopharyngioma patients will be a priority in the future. Identification of those at highest risk for hypothalamic obesity and intervention with either bariatric procedures or pharmacological therapy before the development of morbid obesity has the potential to reduce morbidity.^17,19^

## Conclusion

Management of patients with craniopharyngioma has always been a challenge. The role of the endocrinologist has now expanded to meet the needs of these patients beyond the area of hormone replacement in a multidisciplinary and patient-orientated approach. The pooling of data between centres and the development of clinical care recommendations, both within Europe and beyond, will be important for the future well-being of craniopharyngioma patients.
